# A new therapy for highly effective tumor eradication using HVJ-E combined with chemotherapy

**DOI:** 10.1186/1741-7015-5-28

**Published:** 2007-09-21

**Authors:** Hirokazu Kawano, Shintarou Komaba, Toshihide Kanamori, Yasufumi Kaneda

**Affiliations:** 1 Division of Gene Therapy Science, Osaka University Graduate School of Medicine, 2-2 Yamada-oka, Suita, Osaka 565-0871, Japan; 2 GenomIdea Inc., 7-7-15 Saito-Asagi, Ibaraki, Osaka 567-0085, Japan

## Abstract

**Background:**

Inactivated HVJ (hemagglutinating virus of Japan; Sendai virus) particles (HVJ envelope vector; HVJ-E can incorporate and deliver plasmid DNA, siRNA, antibody and peptide and anti-cancer drugs to cells both *in vitro *and *in vivo*. We attempted to eradicate tumors derived from mouse colon cancer cells, CT26, by combining bleomycin (BLM)-incorporated HVJ-E (HVJ-E/BLM) with cisplatin (CDDP) administration.

**Methods:**

CT-26 tumor mass was intradermally established in Balb/c mice. HVJ-E/BLM was directly injected into the tumor mass with or without intraperitoneal administration of CDDP. The anti-tumor effect was evaluated by measuring tumor size and cytotoxic T cell activity against CT26. Re-challenge of tumor cells to treated mice was performed 10 days or 8 months after the initial tumor inoculation.

**Results:**

We found that three intratumoral injections of HVJ-E/BLM along with a single intraperitoneal administration of CDDP eradicated CT26 tumors with more than 75% efficiency. When tumor cells were intradermally re-injected on day 10 after the initial tumor inoculation, tumors on both sides disappeared in most of the mice that received the combination therapy of HVJ-E/BLM and CDDP. Eight months after the initial tumor eradication, surviving mice were re-challenged with CT26 cells. The re-challenged tumors were rejected in all of the surviving mice treated with the combination therapy. Cytotoxic T lymphocytes specific for CT26 were generated in these surviving mice.

**Conclusion:**

Combination therapy consisting of HVJ-E and chemotherapy completely eradicated the tumor, and generated anti-tumor immunity. The combination therapy could therefore be a promising new strategy for cancer therapy.

## Background

Although surgery, chemotherapy, and radiotherapy have contributed to the successful treatment of cancer, there are still many cases of cancer that are not eradicated by these treatments. The treatment of advanced and metastatic cancers and the prevention of recurrence are the most difficult problems for cancer therapy.

Oncolytic viruses have received attention from researchers because of their powerful killing effect on cancer cells [1,2]. Some viruses have been discovered from viral mutants [3] or constructed by viral genome engineering [4,5]. The oncolytic viruses have worked very well in animal tumor models. However, they have been less successful in the treatment of humans [6]. Moreover, tumor-selective replication of the viruses is not strict enough to prevent viral replication in non-cancerous cells [7], although the efficiency of replication is much lower than that in cancer cells.

To minimize the side effects of oncolytic viruses, we have been focusing on the anti-tumor activities of inactivated HVJ particles (HVJ-E). We recently found that HVJ-E itself mediates a powerful anti-tumor effect by enhancing cytokine production in dendritic cells (DCs), generating tumor-specific cytotoxic T cells (CTLs), and inhibiting regulatory T-cell activity [8]. However, direct tumor-killing by HVJ-E was not detected in murine tumors.

HVJ-E was originally developed as a novel drug delivery system [9]. We tested the feasibility of HVJ-E vector for the delivery of anticancer reagents and found that bleomycin (BLM), an anticancer antibiotic, could be delivered to cancer cells by the HVJ-E vector approximately 300-fold more effectively than by BLM alone [10]. Taken together, our data suggest that HVJ-E enables the creation of a vector system with the dual functions of drug delivery and immunostimulation. However, in Mima's report [10], the HVJ-E containing bleomycin (HVJ-E/BLM) was administered into intraperitoneal cavity disseminated with colon cancer cells. The anti-tumor effect of HVJ-E/BLM on solid tumors remains to be examined.

In the present study, we demonstrated that intra-tumor injection of HVJ-E/BLM combined with systemic administration of cis-diamminedichloroplatinum (II) (CDDP; cisplatin) achieved highly effective tumor eradication by both inducing anti-tumor immunity and delivering anti-cancer reagent to tumors.

## Methods

### Preparation of BLM-incorporated HVJ-E vector

HVJ-E vector was prepared as previously described [9]. Briefly, 100 μl of 10000-fold diluted HVJ seed solution was injected into the allantoic cavity of 10-day-old embryonated chicken eggs. After 3 days of incubation, the allantoic fluid was harvested and the titer of recovered virus (live HVJ) was measured in hemagglutination units (HAU). An HVJ suspension (6000 HAU) was inactivated by irradiation (99 mJ/cm^2^) and mixed with 60 μl of 40 mg/ml bleomycin (BLM) and 2 μl of 3% Triton X-100. After incubating for 15 min at 4°C, the suspension was washed with 500 μl of saline (Otsuka, Tokyo, Japan) and centrifuged (15000 *g*) for 15 min at 4°C to obtain HVJ-E vector. Then, the suspension was washed an additional two times with 500 μl of saline to completely remove the detergent and the unincorporated BLM. After centrifugation, the HVJ-E/BLM was suspended in 180 μl of saline. The efficacy of BLM inclusion into the vector was quantitatively measured with HPLC. The amount of BLM in 1000 HAU of HVJ-E/BLM was 0.18 μg when 5 mg/ml BLM was used, 0.41 μg with 10 mg/ml BLM, 0.84 μg with 20 mg/ml BLM, 1.12 μg with 30 mg/ml BLM, 1.32 μg with 40 mg/ml BLM, 1.34 μg with 50 mg/ml BLM and 1.52 μg with 100 mg/ml BLM. Thus, the amount of BLM incorporated into the HVJ-E vector increased in proportion to the BLM concentration up to 40 mg/ml of BLM and plateaued at BLM concentrations greater than 40 mg/ml. The 40 mg/ml concentration of BLM was used to prepare the HVJ-E/BLM used in these experiments.

### *In vitro *experiments

CT-26 cells (American Type Culture Collection, Manassas, VA, USA) were maintained in DMEM (Nakalai Tesque, Kyoto, Japan) supplemented with 10% fetal bovine serum, penicillin (50 units/ml) (Nakalai), and streptomycin (50 μg/ml) (Nakalai) and incubated at 37°C in a 5% CO_2_. HVJ-E/BLM was prepared with 40 mg/ml of BLM diluted with saline. BLM (BLEO) was purchased from Nippon Kayaku (Tokyo, Japan).

### *In vivo *experiments

All animal experiments were approved by the Animal Committee of Osaka University and conducted in a humane fashion according to their guidelines. Male BALB/c mice, 5–6 weeks of age, were obtained from Charles River Japan, Inc (Yokohama, Japan). Mice were housed for 7 days and allowed free access to food and water. For tumor cell implantation, CT-26 cells were enzymatically detached from culture flasks and counted. Viable cells (5 × 10^6^) were resuspended in 100 μl of PBS and intradermally (i.d.) injected into the right flanks of the mice. Five days after tumor inoculation, intraperitoneal (i.p.) administration of 200 μg of CDDP (Platosin Inj 10, Pfizer, Tokyo) and/or intratumoral (i.t.) injections of 100 μl of HVJ-E/BLM (5000 HAU) (day 1 and/or 6 day and/or 9 day after CDDP injection) were administrated. After injection of CDDP, tumor size was measured every 3 days, and survival of the animals was monitored. Tumor volume was calculated using the following formula: tumor volume (mm^3^) = length × (width)^2^/2.

### Cytotoxic T lymphocyte (CTL) assay

Spleen cells were harvested 10 days after CDDP administration. The spleen cells (5 × 10^6 ^cells/well) were co-cultured with mitomycin C (MMC)-treated tumor cells at a ratio of 20:1 in 24-well plates. Each well contained 2 ml of T-cell culture medium that consisted of RPMI1640 medium supplemented with 10% heat-inactivated FBS, antibiotics, and 50 μM 2-mercaptoethanol. Cells were cultured at 37°C in 5% CO_2_. These cells, which contained CTLs, were harvested after 5 days of culture and used as effector cells in a standard 4-h ^51^Cr release assay to examine the cytolytic activity of effector cells against target tumor cells. Briefly, target tumor cells (1 × 10^6^) were labeled with 100 μCi of ^51^Cr (Amersham Bioscience, Buckinghamshire, UK) in 200 μl of RPMI1640 supplemented with 10% FBS for 90 min at 37°C. The labeled target cells (1 × 10^4 ^cells/well) were incubated with the effector cells in 96-well microtiter plates. Cells were incubated in 200 μl of T-cell medium for 4 h at 37°C. Various effector/target (E/T) ratios were used. The plates were then centrifuged, and the radioactivity of the supernatants was counted using a MicroBeta Trilux Scintillation Counter (Wallac, Gaithersburg, MD, USA). The maximum release was defined as the counts from samples incubated with 2% Triton X-100, and the spontaneous release was defined as the counts from samples incubated with medium alone. Cytolytic activity was calculated using the following formula: percentage of specific ^51^Cr release = (experimental release - spontaneous release) × 100/(maximum release-spontaneous release). Assays were performed in duplicate wells. The spontaneous release of all assays was <20% of the maximum release.

### Re-challenge experiment

Fifteen days after the CDDP administration that was followed by single or multiple injections of HVJ-E/BLM, the BALB/c mice were re-challenged by intradermal (i.d.) injection of 5 × 10^6 ^CT-26 cells (n = 5) or Meth A cells (n = 5). The injection site was proximal to (but not the same as) the initial injection site. After tumor challenge, the tumor size was measured with calipers every 3 days. The measurements were made in a blinded manner. Tumor volume was then calculated.

Eight months after the tumors were eradicated in four mice (9 months after the initial tumor inoculation), these mice were re-challenged by i.d. injection of 5 × 10^6 ^parental cells into the back.

## Results

### HVJ-E/BLM combined with CDDP treatment can eradicate the established tumor mass

**Figure 1:**
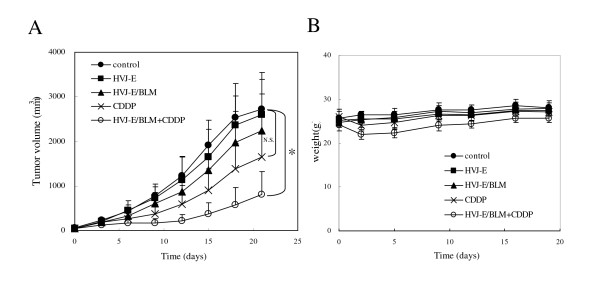
**Effect of HVJ-E/BLM combined with CDDP and body weight**. (A) The effect of HVJ-E/BLM combined with CDDP treatment against CT-26 tumors. When tumors in the dorsa of BALB/c mice reached 5 mm in diameter, HVJ-E (5000 HAU, i.t.), HVJ-E/BLM (5000 HAU, i.t.), CDDP (i.p.), or CDDP (i.p.) plus HVJ-E/BLM (5000 HAU, i.t.) was administered. The tumor diameter was measured every 3 days. Results are expressed as the mean (n = 8 per group). Data are representative of each group. Two independent experiments were performed. CDDP and HVJ-E/BLM treated tumor growth was strongly suppressed compared with saline treated tumors (control). *p < 0.05. By contrast, no significant difference (NS) was seen between control and CDDP alone. Results were statistically analyzed using the Steel-Dwass test. (B) The body weight of each groups. Data are expressed as means ± SD. No significant difference was seen in all groups.

We compared anti-tumor effects of the combination of HVJ-E/BLM and CDDP with HVJ-E/BLM alone or CDDP alone. As shown in Figure 1, 21 days after treatment, the tumor volume of mice treated with HVJ-E containing 1.32 μg BLM was 29.9% less than that of the non-treated control mice, whereas HVJ-E alone resulted in 15.5% suppression in tumor volume. The suppression of tumors injected with 6.5 μg BLM alone was almost same as that with HVJ-E alone (data not shown). With i.p. injection of CDDP without HVJ-E/BLM, mice showed a 46.5% suppression of tumor volume; however, CDDP alone did not eradicate the tumors. The CDDP-mediated decrease in tumor size was transient; eventually all of the tumors continued to grow. By contrast, when HVJ-E/BLM was intratumorally injected one day after CDDP administration, mice had a 68.2% suppression of tumor volume. Remarkably, in 2 of the 10 mice treated with CDDP and HVJ-E/BLM, the tumors were eradicated. After CDDP administration, the body weight of mice treated with CDDP alone or CDDP plus HVJ-E/BLM group decreased transiently, but statistically, no significant difference was seen in the body weight between mice treated with CDDP alone and mice treated with CDDP plus HVJ-E/BLM (Figure 1B).

### CDDP and multiple injections of HVJ-E/BLM increase the frequency of tumor eradication

**Figure 2:**
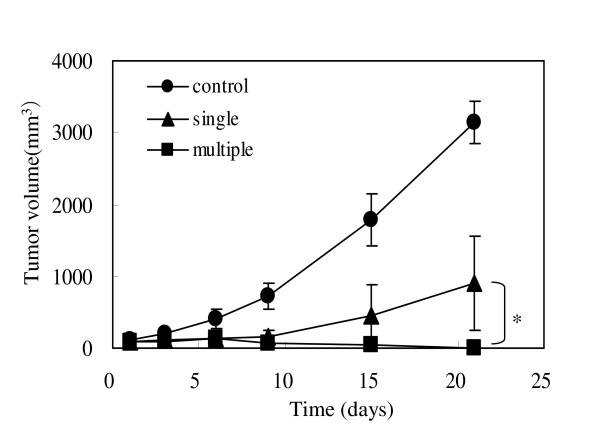
**The effect of multiple injections of HVJ-E/BLM on tumor suppression**. Multiple injections of HVJ-E/BLM were administered. The effect of an i.p. injection of CDDP, followed by a single injection or multiple injections of HVJ-E/BLM on days 1, 6, and 9 was investigated. Results are expressed as means ± SD (n = 4 per group). p < 0.05, single vs multiple injection, Student's *t *test.

Considering that HVJ-E is a potent immune adjuvant [8], multiple injections of HVJ-E can be more effective for tumor suppression by eliciting anti-tumor immunity. We compared the tumor suppression mediated by multiple i.t. injections of HVJ-E/BLM with that mediated by a single injection of HVJ-E/BLM. In both cases, mice received a single i.p. CDDP treatment. Three repeated i.t. injections of HVJ-E/BLM into the established CT-26 tumors were performed on days 1, 6, and 9 after a single i.p. injection of CDDP on day 0. After 21 days, the tumors in one of four (25%) mice that received a single injection of HVJ-E/BLM had been eradicated. Multiple injections of HVJ-E/BLM increased the percentage of tumors that were eradicated to more than 75% (three of four mice in this experiment, four of five mice and four of four mice in two other experiments) (Figure 2), indicating that multiple injections of HVJ-E/BLM were extremely effective in eradicating tumors.

### Multiple injections of HVJ-E/BLM induce the eradication of not only primary tumor but also secondary tumor

**Figure 3:**
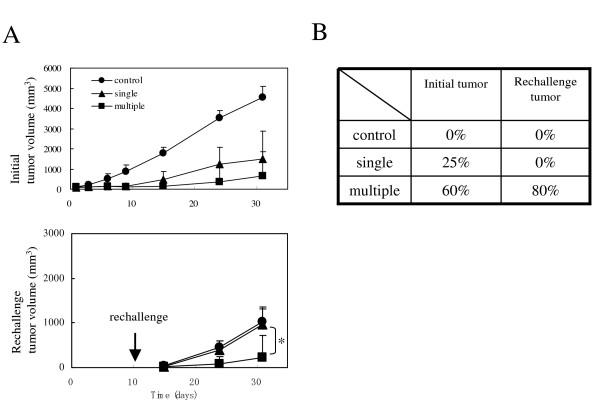
**Tumor volume and eradication rate**. (A) Tumor volume of re-challenged parental CT-26 cells. Animals that were treated with CDDP and one (single) or three (multiple) injections of HVJ-E/BLM were intradermally re-challenged with 5 × 10^6 ^parental CT-26 cells on day 10 after CDDP administration (arrow). The initial (upper) and re-challenged (lower) tumor volumes per mouse were assessed (mean value ± standard deviation). p < 0.05, single vs multiple injection, Student's *t *test. The control mice were age-matched mice that were intradermally inoculated with CT-26 cells. (B) Eradication rate of initial or re-challenge tumor. On day 31 after CDDP administration (on day 16 after re-challenge) the tumors – whether visible or invisible – were examined.

We assessed the CT-26 tumor cell re-challenge experiment. Ten days after CDDP treatment, CT-26 cells were intradermally injected into the trunk at a site opposite to the initial injection site. The initial tumor was eradicated in 25% of mice (one of four) that received a single injection and 60% of mice (three of five) that received multiple injections. The re-challenge tumor was not rejected in the single-injection mice and the control mice; however, 80% of the mice (four of five) that received multiple injections rejected the re-challenge tumor on day 20 after the re-challenge (Figure 3). The re-challenge tumor mass in the single-injection mice was nearly identical to the tumor mass in the control mice; however, the tumor mass was suppressed in the multiple-injection mice (Figure 3). These data show that multiple injections of HVJ-E/BLM after CDDP treatment are able to eradicate the initial tumor and also reject the re-challenge tumor.

**Figure 4:**
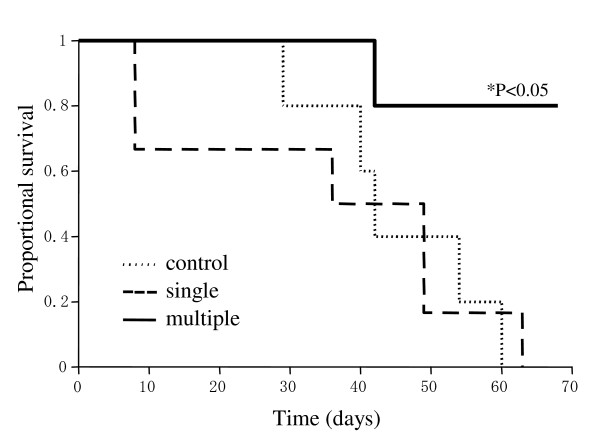
**Survival rate of mice that received a single injection or multiple injections of HVJ-E/BLM**. The mice were injected with HVJ-E/BLM on day 1 or days 1, 6, and 9 after CDDP treatment (i.p.) on day 0. Control mice had no treatment after CT-26 tumor injection. Results are representative of two independent experiments. Results were statistically analyzed using Kaplan-Meier method and log-lank test (n = 5).

Therefore, we assessed the survival rates of mice treated with CDDP plus HVJ-E/BLM (one or three injections) (Figure 4). Tumors were eradicated in 80% of mice that received CDDP and multiple injections of HVJ-E/BLM. No recurrence of tumors was observed in those mice for 8 months. Control mice and the mice treated with a single injection of HVJ-E/BLM and CDDP ultimately succumbed to the CT-26 tumor. The mice able to eradicate their CT-26 tumors survived for a prolonged time period, whereas the control mice and the mice treated with single injection of HVJ-E/BLM and CDDP were all dead by day 63 after intra-dermal inoculation of CT-26 tumor cells. The eradication rates of established tumors were markedly increased by the combined treatment of CDDP and multiple injections of HVJ-E/BLM. Consequently, the survival times of mice receiving CDDP and multiple HVJ-E/BLM injections were significantly prolonged.

### Immune response against CT-26 tumor cells is maintained

**Figure 5:**
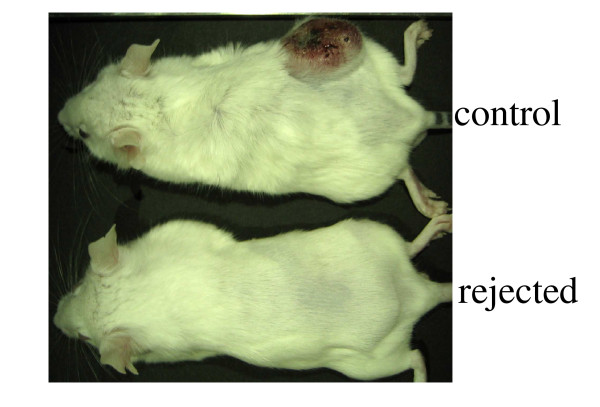
**Long-term anti-tumor immunity**. Mice that rejected the initial i.d. tumor derived from CT-26 cells were re-challenged intradermally with 5 × 10^6 ^parental cells 8 months after the initial tumor eradication (9 months after the initial tumor inoculation). Tumor growth was observed after 30 days (upper: a control mouse; lower: a mouse that previously eradiated the initial tumor). n = 2.

The mice were challenged with CT-26 cells 8 months after they had eradicated the primary CT-26 tumor As shown in Figure 5, the age-matched control mice were uniformly susceptible to this CT-26 challenge. However, the mice that rejected the initial tumor were resistant to the CT-26 i.d. challenge at 8 months after the initial tumor eradication (Figure 5). The mice that eradicated the CT-26 cells could not eradicate the Meth-A cells from a different cell line (data not shown). These results indicate that the combination of CDDP and multiple injections of HVJ-E/BLM induced long-term anti-CT-26 immunity.

**Figure 6:**
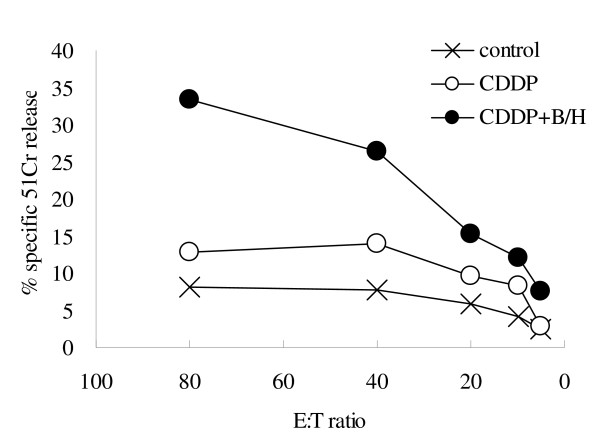
**The induction of CTLs by injection of CDDP and/or HVJ-E/BLM**. When tumors were 5 mm in diameter, CDDP was i.p. injected and HVJ-E/BLM was injected on days 1, 6, and 9 after CDDP treatment (CDDP+B/H). On day 10, mice were sacrificed and splenocyte cells were re-stimulated with CT-26 cells for 5 days, and ^51^Cr release was subsequently assayed. Results are expressed as means (n = 2 per group), representative of two independent experiments. E:T ratio indicates the effector cell to target cell ratio.

To examine anti-tumor immunity, we investigated whether treatment with CDDP plus multiple injections of HVJ-E/BLM induces MHC class I-restricted CT-26-specific CTLs. CTL activity against CT-26 cells was examined in the splenic lymphocyte samples isolated from mice treated with CDDP plus HVJ-E/BLM. After *in vitro *re-stimulation of the splenic lymphocytes with CT-26 cells, we only found a CT-26-specific CTL response in mice that were treated with CDDP plus multiple injections of HVJ-E/BLM (Figure 6). Thus, the multiple i.t. injections of HVJ-E/BLM in combination with a single i.p. CDDP administration could not only eradicate the established CT-26 tumor but also induced long-term immune memory against CT-26 cells in mice.

## Discussion

In this study, we demonstrate that multiple i.t. injections of HVJ-E/BLM combined with a single i.p. administration of CDDP was the most effective treatment for eradicating CT26 tumors in most of the tumor-bearing mice. It appears that this combination therapy induced tumor-killing by chemotherapy and anti-tumor immunity induced by multiple injections of HVJ-E. In chemotherapy, HVJ-E/BLM significantly suppressed tumor growth, but systemic administration of CDDP was indispensable for effective killing of tumors.

We recently reported that HVJ-E itself induced a remarkable infiltration of DCs, CD4^+ ^T cells, and CD8^+ ^T cells into tumors and activated tumor-specific CTLs [8]. Moreover, by inducing the secretion of IL-6 from DCs, HVJ-E inhibited regulatory T cells, which suppress effector T cells [8]. In our current study, multiple HVJ-E/BLM injections into initial tumors suppressed the growth of tumors subsequently implanted on the opposite side of the mouse, and mice showing a complete response rejected a re-challenge with the same tumor cells. CTLs against CT26 were highly activated in the tumor-free mice. These results indicate that multiple injections of HVJ-E/BLM into primary tumors can eradicate metastatic tumors and mediate prophylactic activity against initial tumors.

In our recent paper [8], HVJ-E alone eradicated CT26 tumors, while, as shown in Figure 1, HVJ-E alone was less effective than HVJ-E/BLM. In the former case, we injected 5 × 10^5 ^cells per mouse. However, in the present study, 5 × 10^6 ^CT26 cells were intradermally injected into one mouse. The growth rate of tumors was quite different between both cases. In the former case, on day 20, the tumor reached approximately 600 mm^3^, but in our present study, the volume was 2700 mm^3^. Thus, the tumor model was much more aggressive in our current study than in the previous one. HVJ-E/BLM effectively eradicated tumors even in such an aggressive tumor model, whereas HVJ-E alone failed in tumor regression. Therefore, HVJ-E/BLM is obviously superior to HVJ-E alone in terms of therapeutic efficacy.

Although anti-tumor immunotherapy holds great promise, there have been many obstacles to its development [11,12]. Although current immunotherapy can reduce, delay, or prevent tumor recurrence, many tumors still progress because of the development of mechanisms to avoid recognition and elimination by the immune system [13,14]. The mechanisms of immune system evasion include down-regulation of antigen-processing components and antigen-presentation machinery, weak priming of tumor immunity, and the induction of T-cell tolerance by immunosuppressive molecules produced from cancer cells [15-18]. These problems are a considerable challenge to successful cancer therapy. Therefore, to produce more effective cancer immunotherapy, tumor volume should be reduced as much as possible. Indeed, immunotherapy is often combined with other therapy such as surgical resection, radiotherapy, and chemotherapy [19,20]. Chemotherapy and radiotherapy have been frequently used for the treatment of inoperable tumors. However, chemotherapy and radiotherapy often weaken anti-tumor immunity due to the suppression of bone marrow stem cell function [21]. To increase cytotoxicity of anti-cancer drugs with minimal damage to non-cancerous tissues, new drug delivery systems have been considered. For this purpose, numerous synthetic vectors such as liposomes and micelles have been developed [22]. With these vectors, anti-cancer drugs can be delivered to tumors much more effectively than by circulatory delivery alone [23]. A drug-delivery vector with adjuvant properties is the most appropriate system to achieve both direct tumor killing and anti-tumor immunity induction. Consequently, HVJ-E seems to be an ideal vector system. Furthermore, it is likely that the anti-tumor immunity induced by HVJ-E can be retained even when combined with chemotherapy.

We have already reported anti-tumor effects of HVJ-E in other murine tumor models. Renal cancers transplanted to Balb/c mice intradermally were completely eradicated by three times injection of HVJ-E alone [24] and orthotopic bladder carcinoma was suppressed by intravesical injection of HVJ-E with doxorubicin[25]. Activation of anti-tumor immunity was detected in both cases after HVJ-E treatment.

In a human xenograft tumor model, T cell-mediated anti-tumor immunity is not induced. However, the growth of HeLa cell tumors in SCID mice were dramatically inhibited by intratumoral injection of HVJ-E containing Rad51 siRNA with intraperitoneal administration of cisplatin [26]. Thus, in a human xenograft tumor model, HVJ-E can suppress tumors by the ability of drug delivery.

Treatment with CDDP plus multiple injections of HVJ-E/BLM enhances therapy against primary tumors and metastatic lesions as well as prophylactic activity against tumor recurrence.

We have developed the technologies to produce large amounts of HVJ and to purify HVJ-E and established a pilot plant to produce clinical grade vectors. We will proceed to clinical trials to treat cancers using HVJ-E in a few years.

## Competing interests

The author(s) declare that they have no competing interests.

## Authors' contributions

HK participated in the design of the study, performed the experiments, and drafted the manuscript. SK participated in design of the study, performed the experiments. TK performed the experiments. YK developed drug delivery methods, participated in design of the study and coordination, and critically revised the draft. All authors have read and approved the final manuscript.

## Pre-publication history

The pre-publication history for this paper can be accessed here:


